# Depression Mediates the Relationship between Childhood Trauma and Internet Addiction in Female but Not Male Chinese Adolescents and Young Adults

**DOI:** 10.3390/jcm10215015

**Published:** 2021-10-28

**Authors:** Xue Dong, Ruxin Zhang, Simon Zhornitsky, Thang M. Le, Wuyi Wang, Chiang-Shan R. Li, Sheng Zhang

**Affiliations:** 1 Youth Mental Health Education Center, Department of Psychology, Shaanxi University of Science & Technology, Xi’an 710021, China; 2 Department of Psychiatry, Yale University School of Medicine, New Haven, CT 06519-1109, USA; simon.zhornitsky@yale.edu (S.Z.); thang.le@yale.edu (T.M.L.); wuyi.wang@yale.edu (W.W.); chiang-shan.li@yale.edu (C.-S.R.L.); 3 Department of Economics and Management, Xi’an University of Technology, Xi’an 710054, China; 1200510001@stu.xaut.edu.cn; 4 Department of Neuroscience, Yale University School of Medicine, New Haven, CT 06519-1109, USA; 5 Interdepartmental Neuroscience Program, Yale University, New Haven, CT 06519-1109, USA

**Keywords:** internet addiction, depression, childhood trauma, adolescents, young adults

## Abstract

Internet addiction is associated with a range of psychological risk factors such as childhood trauma and depression. Studies have also suggested sex differences in internet and other behavioral addictions. However, it remains unclear how childhood trauma, depression and internet addiction inter-relate differently between the sexes. A total of 1749 adolescents and young adults aged 12–27 participated in a survey of sociodemographic characteristics and standardized assessments to evaluate internet addiction (Internet Addiction Test), childhood trauma (Childhood Trauma Questionnaire) and depression (Beck Depression Inventory). Mediation and path analyses were used to examine the relationship between childhood trauma, depression and internet addiction. Internet-addicted females relative to males showed more severe depression but the control participants showed the opposite. Childhood trauma was associated with depression for both internet-addicted males and females; however, internet-addicted females but not males showed significant associations between depression and the severity of internet addiction as well as between childhood trauma and the severity of internet addiction. Further, in females, depression mediated the correlations between all types of childhood trauma and the severity of internet addiction. A path analysis suggested that sexual abuse and emotional neglect contributed most significantly to internet addiction when all types of childhood trauma were examined in one model. The findings suggest sex differences in the relationship between childhood trauma, depression and internet addiction. Childhood trauma contributes to internet addiction through depression only in females. The findings may guide future prevention and intervention strategies of internet addiction.

## 1. Introduction

Internet addiction is defined as the “inability to control internet using, leading to physical, psychological and social difficulties” and is growing in prevalence and severity [1]. The prevalence rate of internet addiction among adolescents and young adults has increased from 1.6% in 2005 [2] to 6.7% in 2010 [3], 10.6% in 2015 [4] and 28.6% in 2020 due to the outbreak of COVID-19 [5,6]. Since the first diagnosis by Young [7] in 1995, internet addiction has received more clinical attention, particularly among adolescents and young adults who have spent significantly more time on smartphones and tablets. Internet addiction has been associated with depression, attention-deficit hyperactivity disorder [8], social anxiety disorder [9], hostility and aggression [10,11] as well as poor academic performance [12]. Although drug addiction distinctly involves physical dependence [13], behavioral addiction—including internet addiction—shares many of the psychological manifestations including craving and withdrawal [14,15].

Adolescents and young adults appear to be the most susceptible to internet addiction [16,17]. The increasing prevalence of internet addiction has been associated with low emotional stability and poor self-regulation in adolescents [18,19]. Adolescents with internet addiction may manifest depression [20] and engage in internet use to cope with emotional distress as well as to seek pleasure, social connections and a sense of achievement [21]. In particular, adolescents spend significantly more time on the internet to establish and maintain social interactions compared with adults [22,23]. Understanding the psychological and social factors that contribute to the severity of internet addiction is of critical importance to public health.

Depression is frequently comorbid with internet addiction in adolescents and young adults [24]. With autoregressive cross-lagged modeling, a recent longitudinal study showed that the severity of internet gaming disorder and depression are reciprocally predictive in college students [25]. Childhood trauma contributes to the development of depression in adolescents [26,27,28]. Childhood trauma generally refers to emotional, physical and sexual abuse as well as emotional and physical neglect [29]. Exposure to childhood trauma is considered to be a major risk factor for psychosocial dysfunction including emotional dysregulation, depression and heightened reward seeking in association with internet addiction [30,31,32]. Earlier studies suggested that childhood physical abuse was a main predictor of internet addiction among high school students [33]. Through the severity of post-traumatic stress disorder, physical and emotional neglect were directly and indirectly associated with internet addiction [34]. More recently, studies have identified depression as a mediator between childhood emotional trauma and internet gaming disorder [35] and between cyber victimization [36] as well as negative life events [37] and internet addiction among adolescents. Further, alexithymia partially mediated the relationship between traumatic experiences and internet addiction among late adolescents [38,39]. Together, these studies suggest depression to be an important mediator linking childhood trauma and internet addiction [40]. However, it remains unclear how depression may mediate the relationship between childhood trauma and internet addiction differently for males and females.

Male adolescents have shown a higher prevalence rate of internet addiction and a more severe internet gaming disorder relative to females [41,42,43]. Conversely, females relative to males demonstrated a more significant association between attention deficits and internet addiction as well as a higher risk of mobile phone addiction [44,45,46]. Other studies have provided evidence for potential sex-specific relations between childhood trauma and addictive behavior. Adolescent females with emotional difficulties such as subjective unhappiness or depressive symptoms had significantly higher risks of internet addiction relative to adolescent males with similar problems [47]. A higher number of traumatic experiences among late adolescent males but not females was predictive of internet addiction [38]. Moreover, female adolescents showed twice the likelihood of developing depression than male adolescents [48,49]. Together, these studies suggest the importance of considering sex differences in investigating the psychological factors of internet addiction and the relationship between childhood trauma, depression and internet addiction.

To this end, we conducted a large-scale cross-sectional study to investigate sex differences in the relationship between childhood trauma, depression and internet addiction in adolescents and young adults. We assessed childhood trauma with the Childhood Trauma Questionnaire (CTQ) and depression with the Beck Depression Inventory (BDI-II) in both internet-addicted and control participants. We employed mediation and path analyses to evaluate how depression may mediate the relationship between childhood trauma and the severity of internet addiction differently in males and females as assessed by the Internet Addiction Test (IAT). We also examined whether specific subtypes of childhood trauma may play a more significant role conducive to depression and internet addiction. 

## 2. Methods

### 2.1. Participants

**Figure 1:**
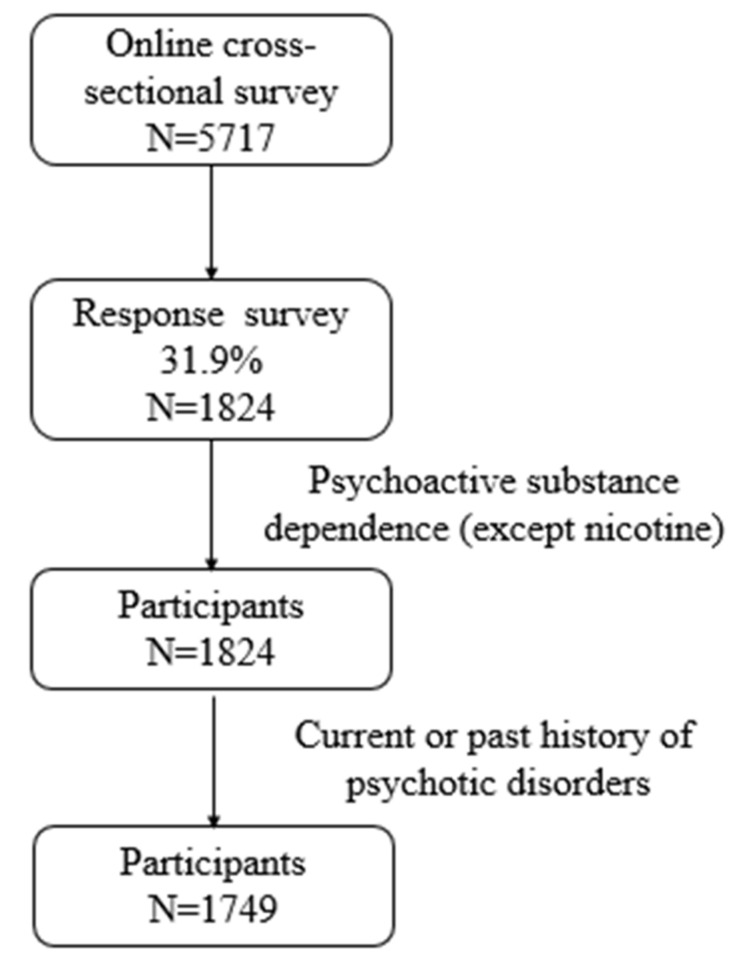
Flowchart of the selection of study participants.

An online cross-sectional survey was conducted on adolescents and young adults in China between March and April 2020 and we invited 5717 regular internet users to participate in our study. A total of 1824 participants took part in the survey and the response rate was 31.9%. We excluded individuals with a dependence on psychoactive substances in order to control the addiction effect other than the internet. However, individuals with nicotine dependence were not excluded as nicotine dependence was common in our samples with and without internet addiction. None of our 1824 participants showed any psychoactive substance use; thus, we did not exclude anyone. Further, we also excluded individuals with a current or past history of psychotic disorders as these disorders are known to have a cognitive and/or emotional dysfunction with unknown effects on our study. Seventy-five participants were excluded from the data analyses because of a current or past history of psychotic disorders. Finally, the data of 1749 participants (877 or 50.1% males) aged 12–27 (18.4 ± 1.9, mean ± SD) years were analyzed in the current study (Figure 1).

All subjects were physically healthy with no major medical illnesses or current use of prescription medications. None reported having a history of a head injury or neurological illness. Sociodemographic characteristics including age, sex, education level, race and academic achievement were included in the survey. Academic achievement was assessed through a self-reported cumulative grade point average ranging from 1.50 to 5.00 for the previous semester, which was used as a categorical variable (Very good: 4.50–5.00; Good: 4.00–4.50; Moderate: 3.50–4.00; Poor: 1.50–3.50). The research was conducted according to a protocol approved by the Ethics Committee of Shaanxi University of Science and Technology. All participants gave their informed consent and parental or guardian consent was obtained for students under 18 years.

### 2.2. Measures of Internet Addiction, Depression and Childhood Trauma

Internet Addiction Test (IAT): The IAT (1998) has 20 items, each rated on a six-point Likert scale ranging from 0 (not at all) to 5 (always), to assess the degree to which internet use affected daily lives. A higher score indicated a higher degree of internet addiction: normal: 0–30; mild: 31–49; moderate: 50–79; and severe: 80–100. This measure has been widely used around the world in studies investigating internet addiction and has been validated in many populations such as the United States [50], French [51], German [52], Polish [53], Spanish [54], Greek [55], Arabic [56], Vietnamese [57], Indonesian [58], Indian [59], Pakistani [60], Thai [61] and Korean [62] as well as Chinese [63]. A more recent review and meta-analysis study suggested that the IAT had an acceptable internal consistency, test-retest reliability and convergent validity among a total of 25 studies including 18,421 subjects [64]. Based on previous studies, we employed a cut-off score of ≥50 to identify individuals with internet addiction according to Young’s criteria [65,66,67,68,69]. The current sample comprised 391 (195 males and 196 females) individuals with internet addiction and 1358 (682 males and 676 females) controls. In this study, the factor loading of each item was between 0.777–0.881 and Cronbach’s α was 0.870, which had a high reliability and validity.

Childhood Trauma Questionnaire (CTQ): A retrospective measure of childhood trauma [70], the CTQ has 28 items to assess traumatic life experiences during childhood and adolescence including emotional abuse, emotional neglect, physical abuse, physical neglect and sexual abuse. Each item is ranked from 1 (never) to 5 (very often) with a higher score indicating a higher severity of childhood trauma. The CTQ has shown an excellent test-retest validity and internal consistency [71].

Beck Depression Inventory (BDI-II): The BDI-II consists of 21 items addressing the symptoms of depression such as weight loss, irritability, hopelessness, suicide ideation, self-guilt and a lack of energy during the previous two weeks [72], each of which has four self-evaluative statements scored from 0 to 3. Greater scores indicate a greater depression severity. The BDI-II scale has been shown to have good internal reliability, consistency and validity [73].

### 2.3. Statistical Analyses

We performed statistical analyses using SPSS 19.0 software. We conducted an χ^2^ test to analyze the race and academic success variables and a two-way ANOVA (grouped by sex) for all other variables. Using MATLAB, we performed a Pearson correction analysis for the BDI, IAT and CTQ scores with age as a covariate and mediation analyses using the toolbox M3. Using AMOS 21.0, we performed path analyses based on structural equation modeling. The details can be found in the “Mediation and Path Analyses” section. The χ^2^ test and ANOVA model as well as the mediation and path analyses were evaluated at *p* < 0.05. The Pearson correction results were evaluated at a corrected threshold of *p* = 0.05/(1 + 6 + 6) = 0.0038 considering a multiple comparison and *p* < 0.05 as a marginal correlation.

### 2.4. Mediation and Path Analyses

We performed mediation analyses [74], using the toolbox M3 (https://github.com/canlab/MediationToolbox/tree/master/mediation_toolbox) to examine how depression mediated the relationship between childhood trauma (CTQ and subscores) and internet addiction. In a mediation analysis, the relation between the independent variable X and the dependent variable Y, i.e., X→Y, was tested to see if it was significantly mediated by a variable, M. The mediation test was performed by employing three regression equations [74]:

$$Y=i_{1}+\mathrm{cX}+e_{1}$$

$$Y=i_{2}+c^{\prime }X+\mathrm{bM}+e_{2}$$

$$M=i_{3}+\mathrm{aX}+e_{3}$$

where a represents X→M, b represents M→Y (controlling for X), c′ represents X→Y (controlling for M) and c represents X→Y. The constants i_1_, i_2_ and i_3_ are the intercepts and e_1_, e_2_ and e_3_ are the residual errors. In the literature, a, b, c and c′ were referred to as the path coefficients or simply the paths [74] and we followed this notation. Variable M is said to be a mediator of the correlation X→Y if (c–c′), which is mathematically equivalent to the product of the paths a × b, is significantly different from zero [74]. If the product a × b and the paths a and b are significant, one concludes that X→Y is mediated by M. In addition, if path c′ is not significant, there is no direct connection from X to Y and that X→Y is completely mediated by M. Note that path b is the relation between Y and M controlling for X and should not be confused with the correlation coefficient between Y and M.

A path analysis was performed to identify the relationships between childhood trauma, depression and internet addiction using structural equation modeling (SEM). SEM is a multivariate analysis to examine structural relationships [75], combining a confirmatory factor analysis with a multiple regression and enabling hypothesis testing about multiple relations among observed and latent variables [76]. SEM has the advantage of simultaneously estimating multiple and mutual dependencies, identifying latent variables for these relationships and returning direct and indirect effect sizes. Importantly, SEM can handle multiple dependent variables simultaneously. As the CTQ subscores were highly correlated, we employed SEM to include the BDI, IAT and all CTQ subscores in one model to account for the inter-relationship among the CTQ subscores. SEM has been widely applied to the research of internet addiction [77,78,79].

## 3. Results

### 3.1. Sociodemographic and Clinical Variables

**Figure 2:**
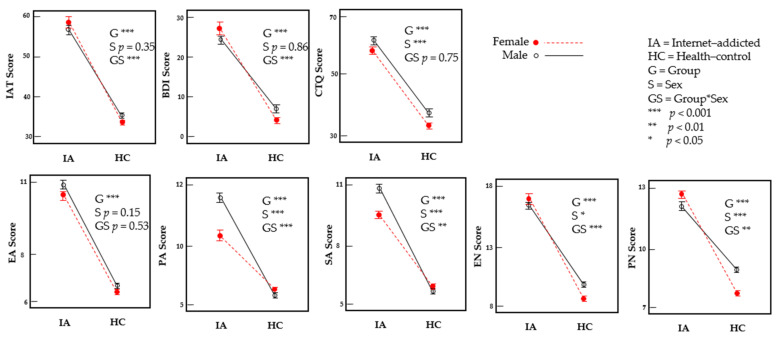
Mean and error bar (SD) plot of the BDI, IAT and CTQ totals and subscores shown separately for internet-addicted males, internet-addicted females, control males and control females. Significant group effects by sex interaction are represented by the cross lines between the internet–addicted males–control males and the internet–addicted females–control females.

The prevalence of internet addiction in this sample was 22.3% (391/1749) based on Young’s criteria of an IAT score ≥ 50. Of the 391 individuals in the internet-addicted group, 49.9% (195/391) were males and 50.1% (196/391) were females. The mean ± standard deviation values of the clinical variables are shown in Figure 2 and the statistics of the group main effect, sex main effect and group by sex interaction effect are shown in Table 1. The internet-addicted vs. the control group exhibited a higher level of the IAT score, BDI score and CTQ total and all subscores (all *p* < 0.001). In the sex main effect, the males showed a higher PA (*p* < 0.001), SA (*p* < 0.001), EN (*p* < 0.05) and PN (*p* < 0.001) but not IAT (*p* = 0.35), BDI (*p* = 0.86) and EA (*p* = 0.15) score compared with females. There was also a significant group by sex interaction for the IAT (*p* < 0.001), BDI (*p* < 0.001), PA (*p* < 0.001), SA (*p* < 0.01), EN (*p* < 0.001) and PN (*p* < 0.01) score but not for the CTQ total score (*p* = 0.75) and EA (*p* = 0.53). In the post-hoc analysis of sex difference, the male vs. the female controls showed a higher level of all variables (all *p* < 0.007). However, internet-addicted males vs. females showed a higher PA (*p* < 0.001) and SA (*p* < 0.001) but a lower BDI (*p* = 0.035) score. There was no difference for the other variables (all *p* > 0.063).

### 3.2. Associations between the BDI, IAT and CTQ Scores in the Internet-Addicted Group

**Figure 3:**
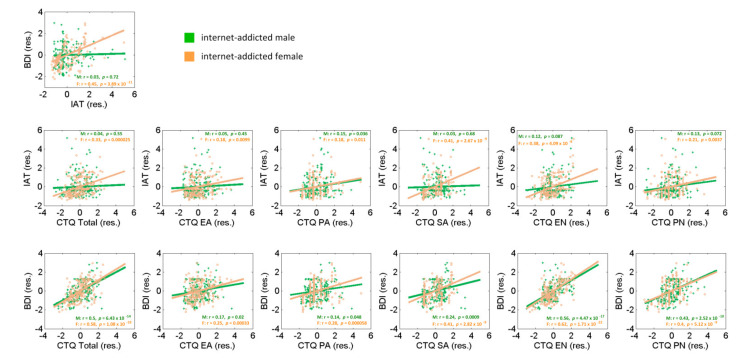
Linear regression of the BDI, IAT and CTQ totals and subscores shown separately for the internet-addicted males and females with age as a covariate. Each data point represents the residual after accounting for age. Green (crosses) and orange (circles) show the data points and regression lines for males and females, respectively.

We computed the Pearson correlations between the BDI, IAT and CTQ scores for the internet-addicted group with age as a covariate (Figure 3) and evaluated the results at a corrected threshold of *p* = 0.05/(1 + 6 + 6) = 0.0038 considering for a multiple comparison. The BDI and IAT scores showed a positive correlation for females (r = 0.45, *p* = 3.89 × 10^−11^) but not males (r = 0.03, *p* = 0.72) and the sex difference of the correlations was confirmed by a slope test (z = −4.46, *p* < 0.0001). For females, the IAT score was positively correlated with the CTQ total (r = 0.33, *p* = 0.000025) and the subscores of SA (r = 0.41, *p* = 2.67 × 10^−9^), EN (r = 0.38, *p* = 4.09 × 10^−8^) and PN (r = 0.21, *p* = 0.0037) as well as at a marginal level for EA (r = 0.18, *p* = 0.0099) and PA (r = 0.18, *p* = 0.011). For males, only the PA subscore showed a marginal correlation with the IAT score (r = 0.15, *p* = 0.036). The slope test showed sex differences for a BDI correlation with the CTQ total (z = −2.97, *p* = 0.003) and SA (z = −3.98, *p* = 0.0001) and at a marginal level for the EN (z = −2.74, *p* = 0.0061) scores but not with the EA (z = −1.29, *p* = 0.2), PA (z = −0.3, *p* = 0.76) and PN (z = −0.81, *p* = 0.42) scores. Further, the BDI score was correlated with the CTQ total and subscores for both females (r = 0.58, *p* = 1.08 × 10^−18^ for the total score; r = 0.25, *p* = 0.00033 for EA; r = 0.28, *p* = 0.000058 for PA; r = 0.41, *p* = 2.82 × 10^−9^ for SA; r = 0.62, *p* = 1.71 × 10^−22^ for EN; and r = 0.4, *p* = 5.12 × 10^−9^ for PN) and males for the total score (r = 0.5, *p* = 6.43 × 10^−14^), SA (r = 0.24, *p* = 0.0009), EN (r = 0.56, *p* = 4.47 × 10^−17^) and PN (r = 0.43, *p* = 2.52 × 10^−10^) as well as at a marginal level for EA (r = 0.17, *p* = 0.02) and PA (r = 0.14, *p* = 0.048). The slope test showed no sex difference (all *p* > 0.062).

### 3.3. Mediation and Path Analyses for Female Internet-Addicted Individuals

**Figure 4:**
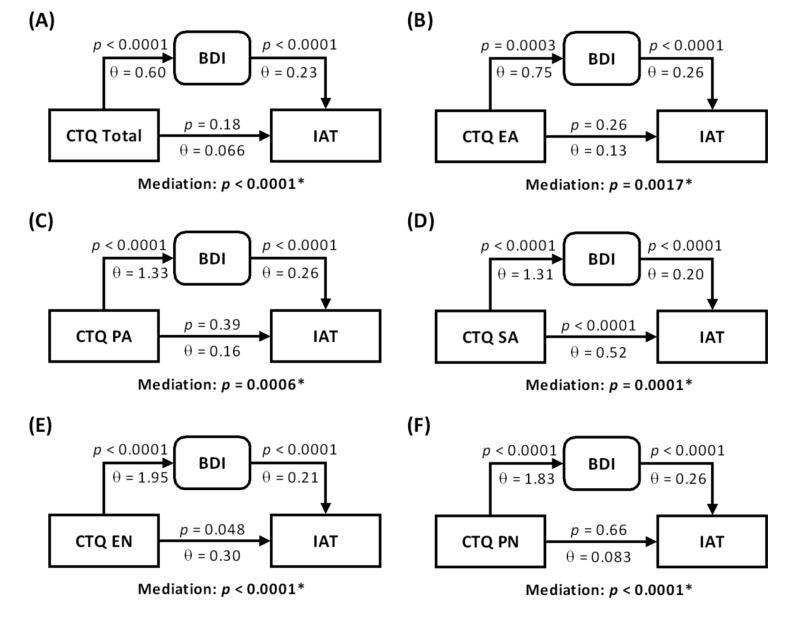
Mediation analysis of the BDI, IAT and CTQ totals (**A**) and subscores (**B**–**F**) in internet-addicted females. The *p*-values associated with mediation are for the path “a × b” (see Section 2.4). All models are significant, suggesting that the BDI mediated the relationship between the CTQ total/subscores and the IAT score. Specifically, the relationships of the CTQ total and the EA, PA and PN scores with the IAT were completely mediated by the BDI score.

As the CTQ, BDI and IAT scores were pair-wise correlated for females with internet addiction, we conducted a mediation analysis to examine whether the BDI mediated the relationship between the CTQ total and five subscores and the IAT. The results showed that all models were significant with the BDI score mediating the correlation between the CTQ and IAT scores (Figure 4). Specifically, without the mediation of the BDI score, the CTQ total, EA, PA or PN were not correlated with the IAT (*p* = 0.18, 0.26, 0.39 and 0.66, respectively) score, suggesting a complete mediation.

**Figure 5:**
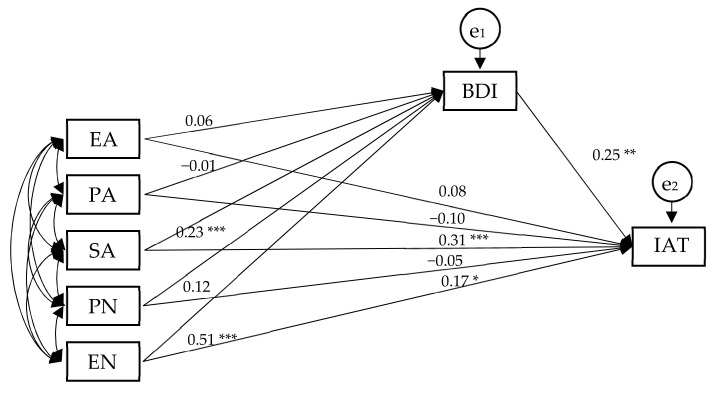
Path analysis of childhood trauma (CTQ subscores), the severity of depression (BDI score) and internet addiction (IAT score) for female internet-addicted individuals. EA = emotional abuse subscale; PA = physical abuse subscale; SA = sexual abuse subscale; EN = emotional neglect subscale; PN = physical neglect subscale. e1 and e2 represent the error terms of the BDI and IAT scores. The value on each path indicates the standardized coefficient. ***: *p* < 0.001; **: *p* < 0.01; *: *p* < 0.05.

We conducted a path analysis to include the BDI score, IAT score and all CTQ subscores in one model for internet-addicted females (Figure 5). The model showed that the BDI (β = 0.25, *p* < 0.01), SA (β = 0.31, *p* < 0.001) and EN (β = 0.17, *p* < 0.05) had significant positive effects on the IAT; SA (β = 0.23, *p* < 0.001) and EN (β = 0.51, *p* < 0.001) had significant positive effects on the BDI (Supplementary Table S1).

Based on the results of the path analysis, we used the Bootstrap method to test the mediation effect of the BDI in internet-addicted females. The result showed significant mediation effects of the BDI in the SA-IAT path (β = 0.11, confidence interval = (0.03, 0.26), *p* < 0.05) and of the BDI in the EN-IAT path (β = 0.24, confidence interval = (0.07, 0.42), *p* < 0.05) (Supplementary Table S2).

## 4. Discussion

We examined sex differences in the relationship between childhood trauma, depression and the severity of internet addiction among Chinese adolescents and young adults. Males showed a greater severity of internet addiction as well as most types of childhood trauma (except for emotional abuse) compared with females. Internet-addicted females showed more severe depression compared with internet-addicted males but the control males showed more severe depression compared with the control females. Childhood trauma was associated with depression for both internet-addicted males and females. However, internet-addicted females but not males showed significant associations between depression and internet addiction and between childhood trauma and the severity of internet addiction. Importantly, depression mediated the correlations between all types of childhood trauma when modeled separately as well as the severity of internet addiction in females. A path analysis further suggested that sexual abuse and emotional neglect contributed more significantly to internet addiction when all childhood trauma subtypes were examined together in one model. The findings suggested sex differences in the relationship between childhood trauma, depression and internet addiction. Childhood trauma is conducive to depression and, in turn, internet addiction only in females.

### 4.1. Childhood Trauma Leads to Internet Addiction through Depression in Females but Not in Males

Childhood trauma contributes to internet addiction in females but not males, despite more severe traumatic experiences of males during childhood. Internet addiction is consistent with childhood trauma among adolescents [80,81]. In addition, sex differences have frequently been observed in the research on the relationship between childhood trauma and addiction. For instance, childhood trauma is associated with a greater severity of gambling problems in pathological gamblers especially female gamblers [82]. A previous study showed that females who experienced childhood emotional neglect, physical and sexual trauma were more likely to develop an alcohol use disorder [83]. Females who reported experiencing child abuse and neglect were significantly more likely to have used illicit drugs compared with males who reported similar childhood trauma [84]. Childhood maltreatment was found to be significantly associated with alcohol problems for females but not males and childhood sexual abuse was found to predict substance abuse in females but not in males [85]. Thus, by showing a female-specific relationship between childhood trauma and internet addiction, the current findings extend this literature of sex differences to behavioral addiction.

Importantly, we observed that childhood trauma contributes to the severity of internet addiction via depression in females. An earlier study showed that childhood trauma had a significant impact on the internet gaming behaviors of adolescents among college students and both anxiety and depression significantly mediated the relationship between childhood trauma and internet gaming [40]. Another study showed that emotion dysregulation and depression mediated the association between adverse childhood experiences and the severity of food and social media addictions [86]. On the other hand, these studies did not examine sex differences and the current findings suggest the importance of considering sex differences in future research.

We showed that, of the subtypes of childhood trauma, sexual abuse and emotional neglect contributed more prominently to internet addiction through depression in females. Studies have reported greater effects of sexual abuse and emotional neglect on the mental health of females [87,88,89,90]. For instance, girls experiencing more severe emotional neglect and childhood maltreatment than boys significantly increased the likelihood of post-traumatic depression for all children but more severely for girls than for boys 89. Childhood emotional neglect and adolescent depression were positively correlated even after controlling for other types of childhood maltreatment and sex moderated the relation between childhood emotional abuse and deviant peer affiliation with the relationship being stronger for girls than for boys [90]. Compared with those who did not experience childhood sexual abuse, females who experienced childhood sexual abuse reported greater levels of alcohol use and anxiety and the anxiety significantly mediated the association between childhood sexual abuse and alcohol abuse [91].

### 4.2. Sex Difference of the BDI and Childhood Trauma

We observed a higher BDI score in internet-addicted females vs. males but a lower BDI score in the control females vs. males. Further, the BDI positively correlated with the IAT score for internet-addicted females but not males. These findings suggested that a higher BDI score as observed in internet-addicted females might represent a consequence of internet addiction. In a three-year longitudinal study of Chinese adolescents, the severity of internet addiction predicted the occurrence of later depression but depression did not predict internet addiction in females whereas the reverse was true in males [92]. Another study showed that individuals with internet addiction vs. controls exhibited more severe depression and the interaction effect of depression by internet addiction was more severe in females than in males [93]. These, along with our findings, suggest that females are more vulnerable to the effects of internet addiction on the pathogenesis and/or progression of depression. Indeed, previous studies have typically found that females experience more depression than males [94,95,96,97] and are more likely to experience negative moods following victimization [98,99,100,101]. Thus, treatment for individuals with internet addiction—particularly females—should involve an effort to prevent the exacerbation of depression.

Previous studies from the United States and Turkey showed that females experienced more emotional and sexual abuse whereas males experienced more physical abuse [102,103,104,105]. Alcohol or other drug-dependent patients showed a higher frequency and higher intensity of childhood trauma compared with the controls and females showed a higher frequency of childhood trauma in all groups compared with males among a Brazilian clinical sample [26]. However, we found that males had higher levels of nearly all types of childhood trauma (except emotional abuse) compared with females, which was consistent with another study of Chinese adolescents [106]. In a large-scale (n = 15,890) cross-sectional survey of the children of rural-to-urban migrant workers in grades 4 to 9 in China, boys were more likely to experience trauma compared with girls [107]. Together, these findings suggest the importance of considering cultural differences in evaluating childhood trauma in adolescents. The culture justifying the use of corporal punishment or verbal reprimands by parents to discipline their sons, for instance, represents a relatively distinct experience of boys growing up in Chinese society [108].

### 4.3. Strengths, Limitations and Conclusions

The current study has three strengths. (1) We conducted a large-scale cross-sectional study to investigate sex differences in the relationship between childhood trauma, depression and internet addiction in Chinese adolescents and young adults; (2) we performed two models to evaluate how depression may mediate the relationship between childhood trauma and the severity of internet addiction and the path analysis model not only cross-validated the findings from the mediation analysis model but also provided additional details by accounting for the inter-relationship among the CTQ subscores; (3) we examined the CTQ total and all subscores in our study.

A few limitations need to be considered for the study. First, the response rate of 31.9% was low. The main reason was that the adolescents and young adults in China were not in school due to COVID-19 when we performed our survey between March and April 2020. We were not able to give them a good explanation about the research and help them to complete the self-administered questionnaires in person. Due to being at home, a few of them may have had a low motivation to participate in our study. On the other hand, nearly half of our data were male (50.1%), which suggested that our dataset was valid to examine the sex difference. Second, there was an age difference between sexes. Although we included age as a covariate in our analyses, we could not entirely rule out the effect of age on the current findings. Our survey was performed during the COVID-19 pandemic and we could not rule out the effect of the pandemic on depression and internet addiction. Thus, future studies are needed for subjects with a matched age between sex with a survey performed during a normal period. Third, the study was limited by the moderate sample size and cross-sectional design. We only explored the relationship between adolescent depression and internet addiction at one point in time. Longitudinal studies are needed to confirm the results of the present study. Fourth, our data comprised self-report items, which are known to be affected by various response biases. Future work should combine information from parents, teachers and psychologists to verify and further explore the relationships. Fifth, the prevalence of severe internet addiction among our sample was only 0.7%. Previous studies found the prevalence rate of severe internet addiction to be 2.5% among high school students in South Korea [109], 2.0% in Japan [110] and 6.44% in first-year university students in China [111]. Thus, one should consider the possibility that the current cohort may not be a representative sample.

In conclusion, the present study is among the first to examine sex differences of the direct and indirect relationships between childhood trauma, depression and internet addiction. The severity of internet addiction of internet-addicted Chinese females but not males was associated with both depression and childhood trauma. Importantly, depression mediated the correlations between all types of childhood trauma and internet addiction. A path analysis further suggested a more significant role of sexual abuse and emotional neglect conducive to internet addiction. Overall, the findings highlighted the critical importance of considering sex as a biological variable when examining the relationship between childhood trauma, depression and internet addiction. In addition, our study confirmed the theoretical model of the effect of childhood trauma on internet addiction and highlighted that depression was an important trigger linking childhood trauma and internet addiction in females but not in males. Our findings have practical and important implications for managers and educators. Managers/educators may closely monitor the depressive state of their female employees/students with childhood trauma in order to reduce internet addiction. Our findings may support and guide more effective health promotion strategies in the treatment of internet addiction.

## Figures and Tables

**Table 1:** Sociodemographic and clinical variables in the internet-addicted (IA) and control groups.

Characteristic	Male IA (*n* = 195)	Female IA (*n* = 196)	Male Control (*n* = 682)	Female Control (*n* = 676)	Group	Sex	Group * Sex
Age	18.68 ± 1.78	18.01 ± 1.82	18.49 ± 1.94	18.39 ± 1.83	F = 0.81 *p* = 0.37	F = 13.21 *p* < 0.001 ***	F = 7.19 *p* = 0.007 **
Race							
Han ethnicity	178 (91.3%)	188 (95.9%)	633 (92.8%)	627 (92.8%)	χ^2^ = 0.31	χ^2^ = 0.65	χ^2^ = 3.53
Other ethnicity	17 (8.7%)	8 (4.1%)	49 (7.2%)	49 (7.2%)	*p* = 0.575	*p* = 0.419	*p* = 0.317
Academic success							
Very good	18 (9.2%)	7 (3.6%)	54 (7.9%)	46 (6.8%)	χ^2^ = 25.17	χ^2^ = 33.59	χ^2^ = 61.61
Good	57 (29.2%)	64 (32.7%)	261 (38.3%)	276 (40.8%)	*p* < 0.001 ***	*p* < 0.001 ***	*p* < 0.001 ***
Moderate	78 (40.0%)	100 (51.0%)	280 (41.1%)	319 (47.2%)			
Poor	42 (21.5%)	25 (12.8%)	87 (12.8%)	35 (5.2%)			
IAT	57.40 ± 6.53	58.67 ± 6.93	35.91 ± 6.38	34.01 ± 5.07	F = 4496.04 *p* < 0.001 ***	F = 0.86 *p* = 0.35	F = 21.28 *p* < 0.001 ***
BDI	23.88 ± 12.91	26.53 ± 11.87	6.75 ± 9.71	3.92 ± 3.28	F = 1592.42 *p* < 0.001 ***	F = 0.033 *p* = 0.86	F = 30.27 *p* < 0.001 ***
CTQ	61.80 ± 11.56	58.20 ± 11.06	37.16 ± 10.21	33.23 ± 5.87	F = 2263.62 *p* < 0.001 ***	F = 52.18 *p* < 0.001 ***	F = 0.100 *p* = 0.75
EA	10.92 ± 3.79	10.80 ± 3.94	6.70 ± 2.26	6.39 ± 1.79	F = 861.57 *p* < 0.001 ***	F = 2.04 *p* = 0.15	F = 0.400 *p* = 0.53
PA	11.34 ± 3.48	8.89 ± 2.47	5.91 ± 1.72	5.35 ± 1.07	F = 1679.31 *p* < 0.001 ***	F = 190.19 *p* < 0.001 ***	F = 74.97 *p* < 0.001 ***
SA	10.86 ± 4.18	9.48 ± 3.61	5.84 ± 2.19	5.27 ± 1.19	F = 1113.39 *p* < 0.001 ***	F = 48.97 *p* < 0.001 ***	F = 8.50 *p* = 0.004 **
EN	16.31 ± 3.75	16.70 ± 3.70	9.80 ± 3.77	8.45 ± 2.41	F = 1520.52 *p* < 0.001 ***	F = 6.55 *p* = 0.011 *	F = 21.15 *p* < 0.001 ***
PN	12.78 ± 2.94	12.71 ± 2.54	8.91 ± 3.18	7.77 ± 2.54	F = 727.81 *p* < 0.001 ***	F = 13.90 *p* < 0.001 ***	F = 10.67 *p* = 0.0011 **

Values are mean ± SD or the actual number (percentage %); IAT = Internet Addiction Test; BDI = Beck Depression Inventory; CTQ = Childhood Trauma Questionnaire; EA = emotional abuse subscale; PA = physical abuse subscale; SA = sexual abuse subscale; EN = emotional neglect subscale; PN = physical neglect subscale; statistically significant difference: *p* < 0.05 *; *p* < 0.01 **; *p* < 0.001 ***.

## Data Availability

Research data are not shared.

## Supplementary Materials

The following are available online at https://www.mdpi.com/article/10.3390/jcm10215015/s1, Table S1: The SEM results of the female internet-addicted group. Table S2: Bootstrapping indirect effects and 95% confidence interval (CI) for the model pathways in the female internet-addicted group.
