# Supplementary material for: Depression Mediates the Relationship between Childhood Trauma and Internet Addiction in Female but Not Male Chinese Adolescents and Young Adults
Source: J Clin Med. 2021 Oct 28;10(21):5015. doi: 10.3390/jcm10215015 (PMC8584624; doi:10.3390/jcm10215015)
Supplement: Supplementary file 1 [file jcm-10-05015-s001.zip › jcm-1381034-supplementary.pdf]

SUPPLEMENTARY MATERIALS

Supplementary Table S1: SEM results of female IA group.

Path			B	S.E.	C.R.	*P*	β
EA	→	BDI	0.18	0.19	0.94	0.35	0.06
PA	→	BDI	-0.07	0.34	-0.20	0.84	-0.01
**SA**	**→**	**BDI**	**0.75**	**0.20**	**3.69**	**<0.001**	**0.23**
PN	→	BDI	0.56	0.29	1.93	0.05	0.12
**EN**	**→**	**BDI**	**1.62**	**0.18**	**8.88**	**<0.001**	**0.51**
BDI	→	IAT	0.15	0.05	3.02	<0.01	0.25
EA	→	IAT	0.15	0.13	1.14	0.25	0.08
PA	→	IAT	-0.28	0.23	-1.24	0.22	-0.10
**SA**	**→**	**IAT**	**0.60**	**0.14**	**4.29**	**<0.001**	**0.31**
PN	→	IAT	-0.13	0.19	-0.68	0.49	-0.05
EN	→	IAT	0.32	0.15	2.20	0.03	0.17

Note: B = unstandardized coefficient; SE = standard errors; CR = t value; β = standardized coefficient;

Supplementary Table S2: Bootstrapping indirect effects and 95% confidence interval (CI) for the model pathways in females IA group

Paths	BootEffect	Bootse	Boot Lower Bounds	Boot Upper Bounds
SA→BDI→IAT	0.11	0.06	0.03	0.26
EN→BDI→IAT	0.24	0.09	0.07	0.42

Note: BootEffect = standardized indirect effect; Bootse = bootstrap standard errors;
